# A Fiber Bragg Grating-Based Monitoring System for Roof Safety Control in Underground Coal Mining

**DOI:** 10.3390/s16101759

**Published:** 2016-10-21

**Authors:** Yiming Zhao, Nong Zhang, Guangyao Si

**Affiliations:** 1 Key Laboratory of Deep Coal Resource Mining of the Ministry of Education, School of Mines, China University of Mining and Technology, Xuzhou 221116, China; zhangnong@cumt.edu.cn; 2 Department of Earth Science and Engineering, Royal School of Mines, Imperial College, London SW7 2AZ, UK; g.si11@imperial.ac.uk

**Keywords:** FBG sensor, safety control, roof, monitoring system, underground coalmine

## Abstract

Monitoring of roof activity is a primary measure adopted in the prevention of roof collapse accidents and functions to optimize and support the design of roadways in underground coalmines. However, traditional monitoring measures, such as using mechanical extensometers or electronic gauges, either require arduous underground labor or cannot function properly in the harsh underground environment. Therefore, in this paper, in order to break through this technological barrier, a novel monitoring system for roof safety control in underground coal mining, using fiber Bragg grating (FBG) material as a perceived element and transmission medium, has been developed. Compared with traditional monitoring equipment, the developed, novel monitoring system has the advantages of providing accurate, reliable, and continuous online monitoring of roof activities in underground coal mining. This is expected to further enable the prevention of catastrophic roof collapse accidents. The system has been successfully implemented at a deep hazardous roadway in Zhuji Coal Mine, China. Monitoring results from the study site have demonstrated the advantages of FBG-based sensors over traditional monitoring approaches. The dynamic impacts of progressive face advance on roof displacement and stress have been accurately captured by the novel roadway roof activity and safety monitoring system, which provided essential references for roadway support and design of the mine.

## 1. Introduction

In recent years, with the depletion of shallow coal resources, coal mining has been extending to deeper and deeper levels, facing ever-increasing stress conditions. This poses a great challenge in the development of roadways, which are an essential part of underground coal mining. High in situ stresses can exacerbate roof subsidence and floor heaving, and can largely increase the costs of roadway support and maintenance. Furthermore, dramatic roof collapse, as a result of excessive roof subsidence and separation, has been recognized as the primary mining hazard in coal mines. This issue can be further complicated by geological anomalies in coal seams, which seriously threaten the safety of miners and limit mineral production. For instance, in China, roof failure accounted for 43% of overall coal mine fatalities in the last decade [1].

Longwall entries, serving an entire longwall panel, are the most common type of roadway in underground coal mines. Compared with tunnels used in civil engineering, this type of roadway has a much shorter lifespan, of about one to two years, but allows for larger deformations. However, the most distinct characteristic of longwall entries is that they are deemed to undergo dynamic stress changes, induced by a progressive longwall face approach. These dynamic stress changes, also referred to as abutment stress, can be as high as four to five times those of in situ stresses in a vertical direction [2]. In order to ensure the roof safety of longwall entries, especially during the period where they are affected by coal extraction activities, roof conditions, such as displacement and stress, are typically monitored. This is expected to provide timely and accurate information on roof stability and reduce the risk of roof failure. However, traditional monitoring techniques, which largely rely on manual measurements, cannot fulfill this purpose. In addition, although electronic gauges have been reported to be successfully applied in tunnels, their implementation under harsh, underground mining conditions is being questioned, as they are prone to interference from electromagnetic signals or can deteriorate due to the high temperatures and moisture content in an underground environment [3].

In recent years, given the unique characteristics of high accuracy, excellent electromagnetic immunity, durability, and distributed measurement, a new sensor, based on fiber Bragg grating (FBG) material as a perceived element and transmission medium, has been widely applied in a variety of industries, such as civil engineering, petroleum engineering, and structural engineering [4,5,6,7,8], especially in harsh environments [9,10]. FBG monitoring in underground engineering has been conducted by many researchers. Huang et al. [11] proposed a novel, distributed self-sensing Fiber Reinforced Polymer (FRP) anchor rod with a built-in optical fiber sensor, which can be used to predict the mechanical behavior of the anchor rod. Based on Bragg sensors, Moerman et al. [12] developed a load cell to measure the forces in ground anchors, and they concluded that fiber-based instrumentation is highly suitable for long-term monitoring purposes. Narus et al. [13] constructed an underground mine monitoring system, based on a Brillouin optical time domain reflectometer (BOTDR) system in the El Teniente Mine (Cachapoal, Chile), and accurately measured the deformation of a ventilation tunnel as a result of stress perturbations induced by a neighboring undercut face. Kim et al. [14] proposed a method enabling FBG sensors to precisely and effectively evaluate the pre-stress forces of pre-stressed concrete wire and strands on actual concrete structures. Viveiros et al. [15,16] presented a fiber optic sensing system and its applications in collecting temperature and gas emission data in harsh underground environments. They remarked on the possible application of using it to detect coal-related fire accidents across the world. Zhao et al. [17] designed a displacement sensor using a fiber optic grating technology, which could be used to monitor tunnel roof displacement in coal mines. Zhou et al. [18] developed an active fiber optic gas sensor to monitor the concentration of a specific gas, which is ideal for detecting explosive or corrosive gases in some practical application scenarios. Liu et al. [19] discussed the potential applications and future trends of fiber optic sensors in coal mine hazard detection and prevention.

Previous studies have provided valuable examples of applying FBG monitoring in different underground engineering problems. Given the appealing advantages of FBG, its application in monitoring roof conditions of longwall entries may contribute significantly to roof management in underground coal mines. However, few efforts have been made to use FBG sensors to investigate the impact of dynamic mining on roof conditions during progressive face approaches.

This paper focuses on developing a FBG-based monitoring system for roof safety control in underground coal mining, which can provide real-time continuous data, including roof displacement and stress loading on roof bolts. Field application of the system in a longwall entry is presented, and the monitoring results are compared with measurement data obtained from traditional techniques.

## 2. Development of the FBG-Based Monitoring System for Roof Safety Control in Underground Coal Mining

### 2.1. Basic Principles of the FBG-Based Monitoring System

In the FBG-based monitoring system, sensors using FBG material act as perceived units for roadway roof activity and changes in mining pressure. One module of a Brillouin optical time domain analysis (BOTDA) interrogator is used as a broadband high-energy light for input in the monitoring network, which consists of FBG sensors that include optical fibers. The BOTDA interrogator is selected since it can provide high accuracy and high quality data along the optical fiber, although it is not the cheapest option. Each FBG sensor reflects a specific peak wavelength of the BOTDA interrogator. During thedrivage of underground roadways, the FBG roof separation and pressure sensors are subjected to strain variations. Consequently, the reflected wavelengths measured by the FBG roof separation and pressure sensors will change accordingly; however, note that not only strain, but also temperature variations, can cause a shift in reflected wavelengths. The response of wavelength variations to strain ε and temperature change △T are given by the following formula [11], which exhibits a linear relationship:

(1) $$\frac{\Delta \lambda }{\lambda_{j}}=(1-P_{e})\varepsilon +(\alpha_{f}+\zeta )\Delta T$$

where Δλ is the shift in reflected wavelength, $\lambda_{j}$ is the specific peak wavelength of the FBG, $P_{e}$ is the effective elasto-optical coefficient of the glass fiber, $\alpha_{f}$ is the thermal expansion coefficient of the glass fiber, and ζ is the thermo-optic coefficient of the glass fiber.

Therefore, in order to obtain a pure strain variation, temperature fluctuations should be eliminated. In this study, an independent FBG sensor, without any exerted stress, was applied to monitor temperature changes. If FBG is only affected by temperature variations, its wavelength variation response to temperature change △T can be described as [20]:

(2) $$\frac{\Delta \lambda_{i}}{\lambda_{i}}=(\alpha_{f}+\zeta )\Delta T$$

where $\Delta \lambda_{i}$ is the shift of the reflected wavelength of the independent FBG sensor and $\lambda_{i}$ is the peak wavelength of an independent FBG sensor.

Substituting Equation (2) into Equation (1), pure strain ε can be expressed as:

(3) $$\varepsilon =(\frac{\Delta \lambda }{\lambda_{j}}-\frac{\Delta \lambda_{i}}{\lambda_{i}})/(1-P_{e})$$

**Figure 1:**
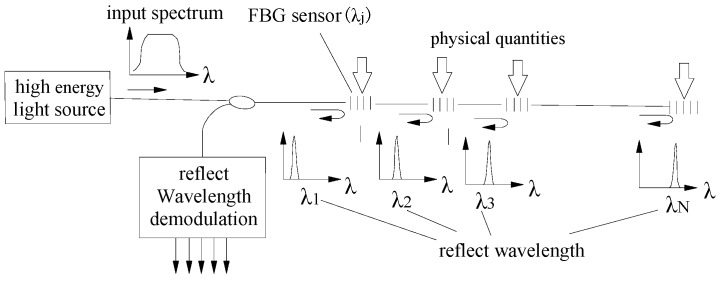
Schematic showing the basic principle of fiber Bragg grating (FBG)-based monitoring sensors.

With the pure strain, the mechanical behavior of roadway roofs, such roof separation and stress loading on roof bolts, can be calculated based on the careful calibration of material properties of the FBG roof separation sensors and FBG stress sensors. The working principle of FBG sensors is depicted in Figure 1.

### 2.2. System Structure

**Figure 2:**
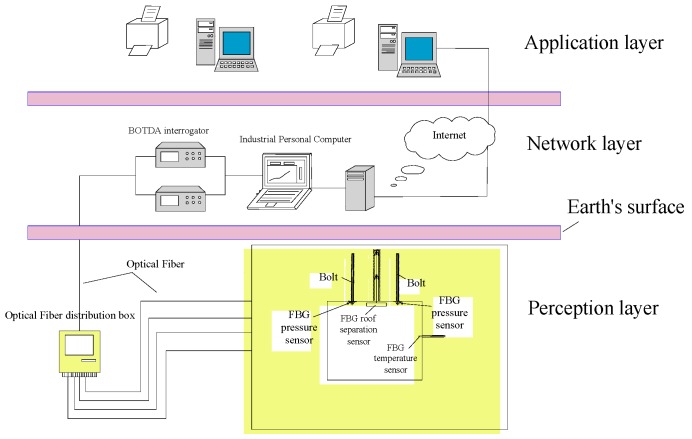
Framework of the FBG-based monitoring system.

According to the basic-level classification methods of the Internet of Things [21], the framework of the FBG-based monitoring system for roof safety control in underground mining consists of the following layers: Perception, Network, and Application, as shown in Figure 2.

#### 2.2.1. Perception Layer

The Perception layer is composed of sensors that use FBG material as a perceived element, including FBG roof separation sensors, FBG temperature sensors, and FBG stress sensors. The FBG sensors are used to detect the values of roof separation and stress loading on roof bolts. After installing these sensors in the roof of the roadway, prior to working face mining, according to monitoring requirements, a complete monitoring network for roof safety control in underground coal mining is formed by the series connection of the sensors.

#### 2.2.2. Network Layer

The Network layer is used to automatically process the data received from the Perception layer. This layer primarily consists of a data acquisition system, data communication and transmission systems, and a data analysis and processing system, all of which can be considered software systems. These systems all have fixed functions, including a real-time display, collection and storage of data, prediction, and Internet access, among others.

#### 2.2.3. Application Layer

The Application layer consists of an industrial-grade personal computer and information exchange network. Users can access the data stored in the hard disk by connecting to the computer via the Internet.

### 2.3. FBG-Based Sensors

#### 2.3.1. FBG Roof Separation Sensor

Roof separation implies that shallow, surrounding rock at an excavation damage zone has been detached from deep stable rock. The detached shallow rock is extremely unstable and poses a potential hazard for miners. FBG roof separation sensors can be used to monitor roof strata separation, up to a maximum separation of 200 mm, which can be used to predict catastrophic roof collapses.

**Figure 3:**
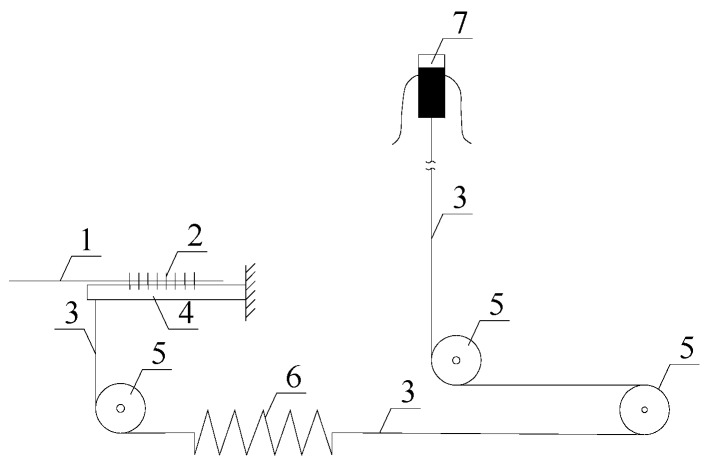
Internal schematic of FBG roof separation sensors: 1—Optical fiber; 2—FBG; 3—Wire rope; 4—Cantilever beam; 5—Fixed roller; 6—Spring; 7—Fixed device.

**Figure 4:**
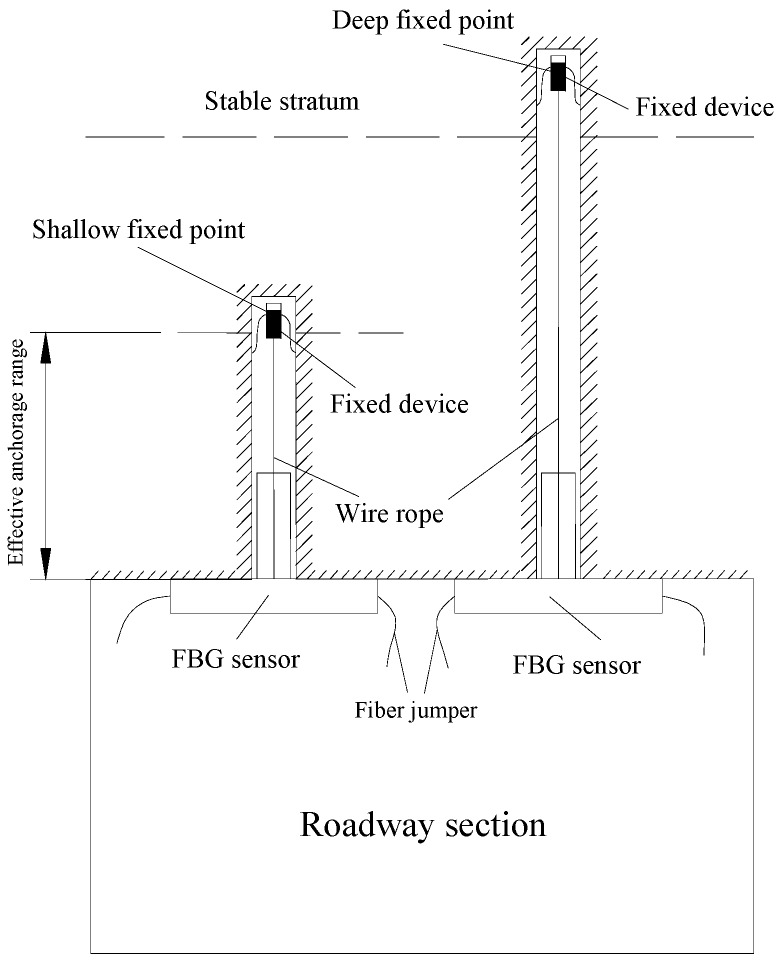
Schematic of FBG roof separation sensors implemented at field.

**Figure 5:**
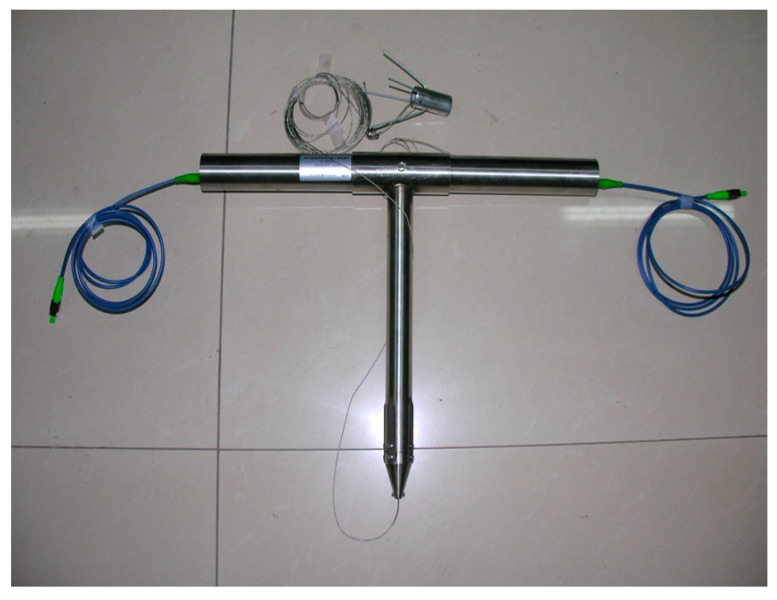
A packaged FBG roof separation sensor.

The internal structural schematic of FBG roof separation sensors is shown in Figure 3. In order to install FBG roof separation sensors, first, the measurement heads have to be fixed at different depths within the roof rock, then the FBG roof separation sensors are connected to the outside openings of the roof boreholes, and, finally, this is followed by the tightening of wire rope. The schematic of this type of sensor implementation is illustrated in Figure 4. In this figure, the left-hand sensor targets measuring roof displacement in shallow surrounding rock (0–3 m to the exaction surface), while the right-hand sensor aims at measuring deep rock deformation. Upon separation of the roof strata, the BOTDA interrogator can detect the strain changes in the reflected wavelength, and converts them into a real-time output of roof displacement. By comparing the displacement differences measured by these two sensors, roof separation can be detected. A packaged FBG roof separation sensor is shown in Figure 5.

#### 2.3.2. FBG Stress Sensor

**Figure 6:**
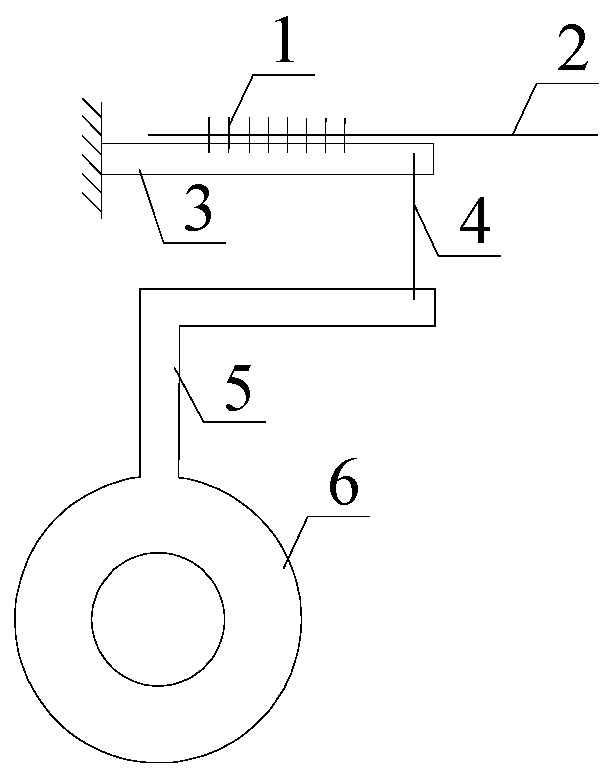
Schematic of FBG stress sensors: 1—FBG; 2—Optic fiber; 3—Cantilever beam; 4—Wire rope; 5—Bourdon tube; 6—pressure ring.

**Figure 7:**
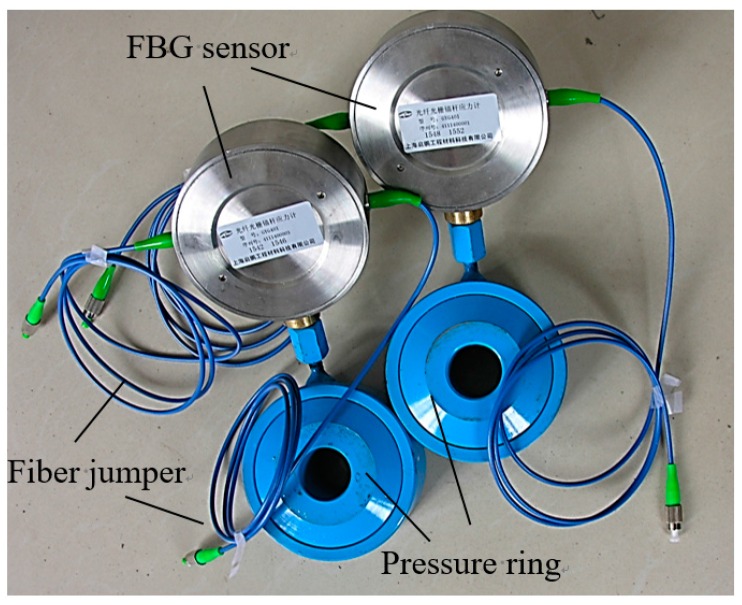
Two packaged FBG stress sensors.

Each FBG stress sensor consists of an FBG sensor, pressure ring, and fiber jumper; the internal structural schematic of this type of sensor is shown in Figure 6, where the maximum value of the FBG stress sensor is 90 MPa. Two packaged FBG stress sensors are shown in Figure 7. The FBG stress sensor should be installed between the support and the surrounding rock, and primarily monitors the resistance exerted by the support on the roadway roof. As long as the roof does not become unsteady, the pressure ring can detect and transmit subtle strain changes to the FBG sensors, and the BOTDA interrogator measures these changes as reflected wavelengths. In doing this, the bolt support resistance can be obtained in real time, and subtle changes in resistance over a long period of time can be used to characterize the stability of a roof.

#### 2.3.3. FBG Temperature Sensor

**Figure 8:**
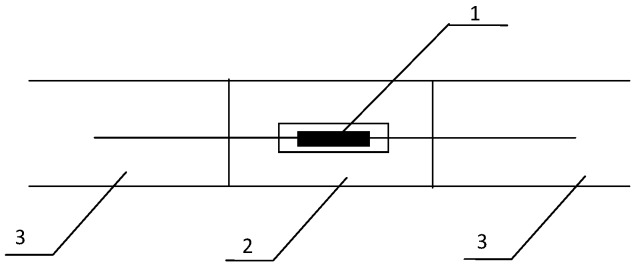
Schematic of FBG temperature sensors: 1—FBG sensor; 2—stainless steel case; 3—Fiber jumper.

**Figure 9:**
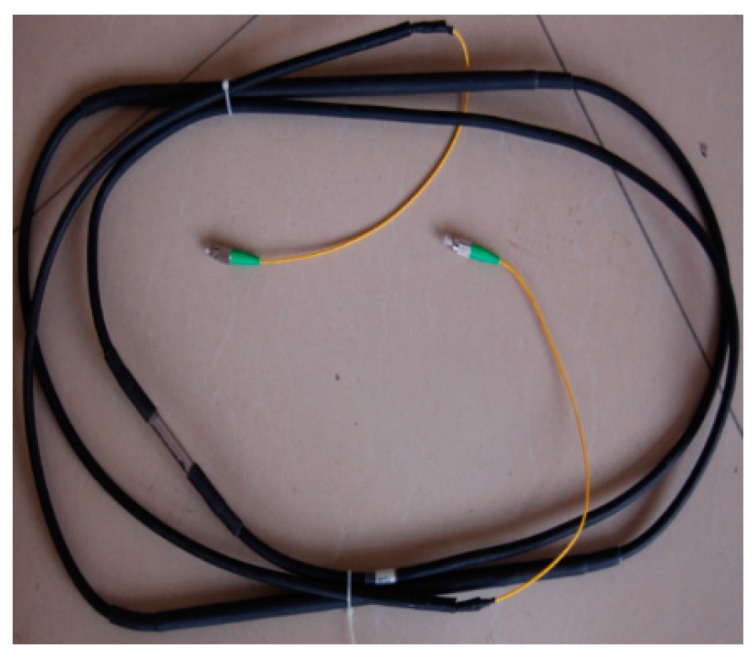
A packaged FBG temperature sensor.

A FBG temperature sensor consists of a FBG sensor, stainless steel case, and fiber jumper, as shown in Figure 8, The FBG temperature sensor is installed at the surface or within the surrounding rock of a roadway to monitor the temperature, and then eliminates the influence of temperature variations on strain measurements. The measurement range is –10 °C to 80 °C, and the measurement accuracy is ±3 °C. A packaged FBG temperature sensor is shown in Figure 9.

#### 2.3.4. Laboratory Calibrations

**Figure 10:**
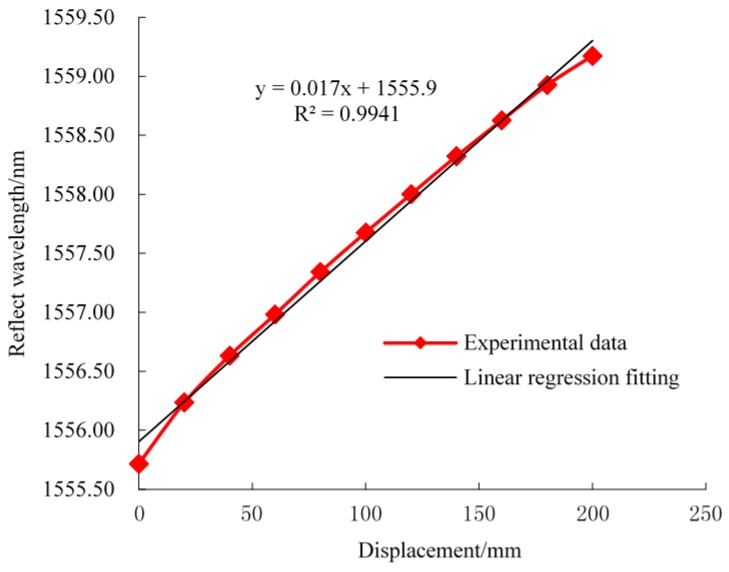
Reflected wavelength and displacement response curve (FBG roof separation sensor).

**Figure 11:**
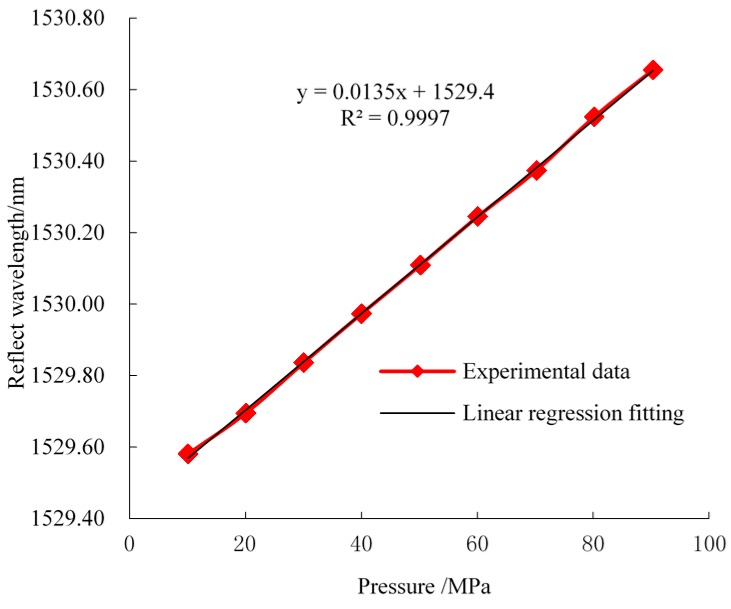
Reflected wavelength and pressure response curve (FBG stress sensor).

**Figure 12:**
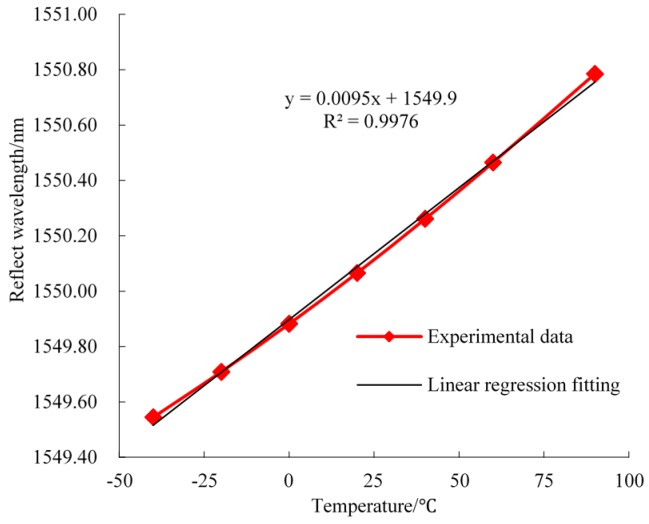
Reflected wavelength and temperature response curve (FBG temperature sensor).

Before being implemented in underground sites, the FBG sensors need to be calibrated under laboratory conditions in order to characterize their mechanical behaviors. Calibration experiments of FBG roof separation sensors and FBG stress sensors were carried out using a material testing machine, which can apply loading pressure to the FBG stress sensors in the range of 0 to 90 MPa and can also control the displacement of FBG roof separation within the range of 0 to 200 mm. A temperature-controlled tank is used to offer an environment with a constant temperature for the FBG temperature sensors. Then, the BOTDA interrogator system is used to monitor FBG reflected wavelength shifts during the process of FBG calibration. Finally, linear relationships of the reflected wavelength and prioritized monitoring of physical quantities were obtained. The laboratory-calibration relationship between the sensor wavelength and the monitoring objectives are shown in Figure 10, Figure 11 and Figure 12. A near-linear relationship can be observed for all three types of sensors, which suggests that a desired accuracy was achieved after the calibration.

## 3. Field Application and Results

### 3.1. Geological Conditions at the Study Site

**Figure 13:**
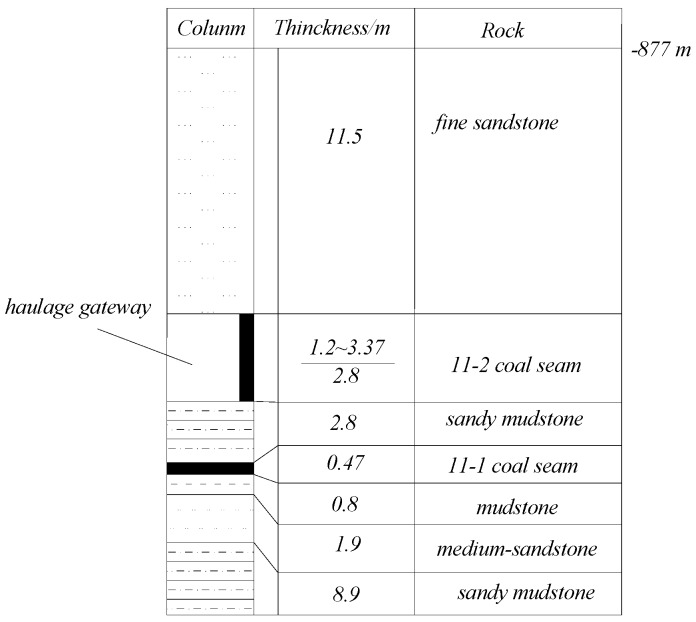
Geological borehole profile of the 1111(1) longwall working face.

The study site was selected as the 1111(1) longwall panel of Zhuji Coal Mine (Huainan Mining Group), China. This longwall panel extracts a 11-2 coal seam, which has an average thickness of 1.31 m and a dip angle of 2°–3°. The 1111(1) longwall face operates 900 m underground, and suffers from extremely high in situ stresses. The in situ stress-measurement results at the mine have suggested that the horizontal stress is approximately 22.73 MPa and the vertical stress is 17.45 MPa. The geological borehole profile of working face 1111(1) is illustrated in Figure 13.

**Figure 14:**
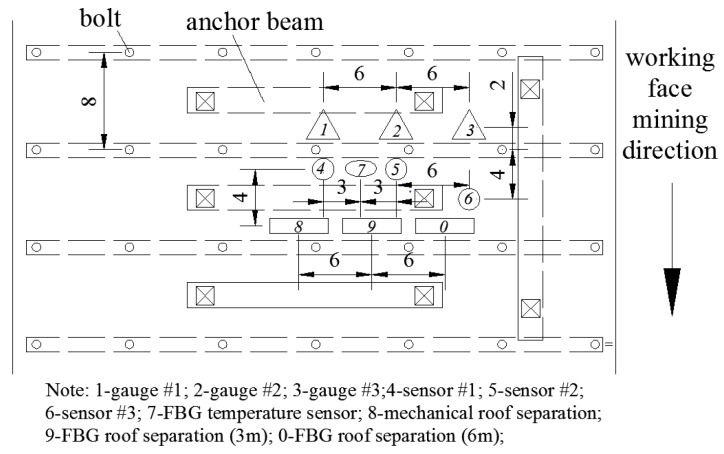
Plan view of FBG sensors layouts in haulage entry roof of 1111(1) working face (unit: Decimeter).

The FBG-based roadway roof activity and safety monitoring system was installed in the haulage entry roof, 250 m ahead of the 1111(1) working face start-line, in order to monitor roof activity and safety during the dynamic advance of the longwall face. The haulage entry of this working face utilizes the gob-side entry retaining structure [22], which is supported by a combination of bolts and cables. The measurements of the haulage entry cross-section are 3 m in height and 5 m in width. The great depth of the coal seam, along with high stress conditions, poses a great challenge in the support and maintenance of this roadway. Furthermore, the unique nature of the gob-side entry requires that its supporting system can withstand the abutment stress, not only induced by the mining of the current panel, but also of the next panel. Therefore, a real-time, continuous stress and roof condition monitoring system is essential for roof safety control at the mine. The layout plan of FBG sensors in the haulage entry roof of the 1111(1) longwall working face is shown in Figure 14. The close distance (less than 1 m) between the FBG temperature sensors and the other FBG sensors ensures that the sensors were all operating roughly under the same temperate conditions and that the calibration of temperature sensors was effective.

### 3.2. Monitoring Scheme

**Figure 15:**
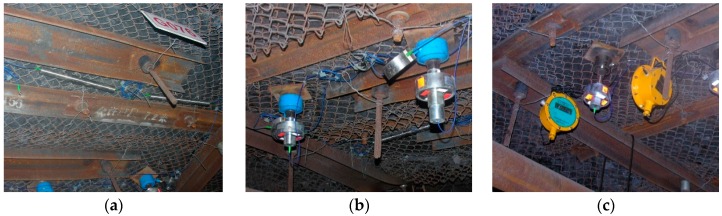
Field implementation of all measurement equipment: (**a**) FBG roof separation sensors; (**b**) FBG stress sensors; (**c**) Electric pressure gauges.

In accordance with the requirements of field monitoring, in the roof of the 1111(1) haulage entry, we installed two FBG roof separation sensors to monitor roof strata activity at depths of 3 m and 6 m, and three FBG stress sensors to monitor bolt axial loading. The maximum frequency of data collection was 1.0 Hz. Simultaneously, traditional mechanical roof separation instruments and electric pressure gauges were also installed for comparison with the FBG sensors. Data from these traditional instruments were manually collected every day. Photos of all measurement equipment in the field, after installation, are presented in Figure 15.

**Figure 16:**
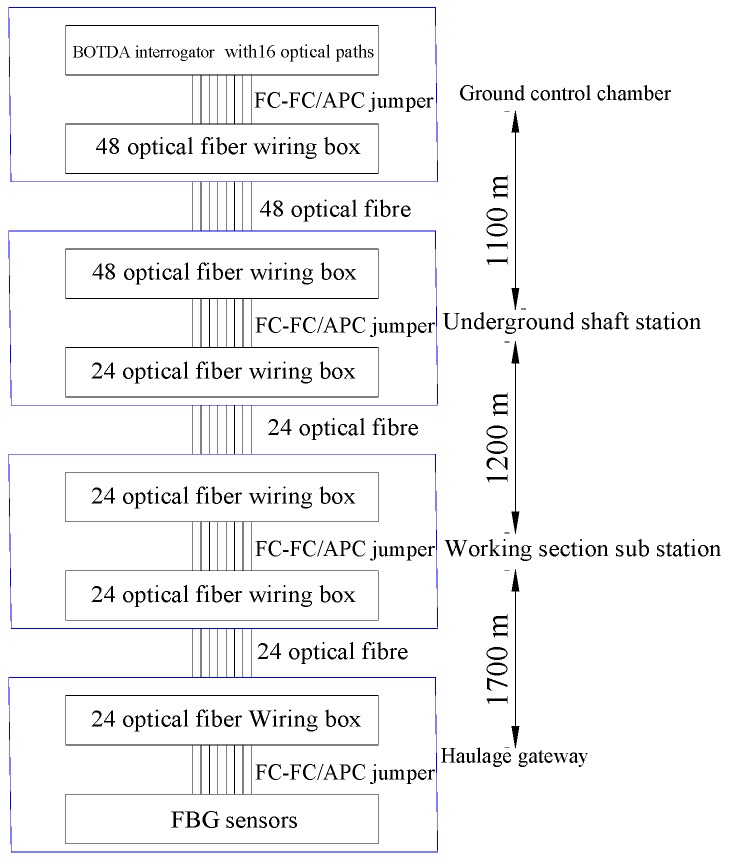
Schematic of the optical path for data transmission at Zhuji coal mine.

Field application of the optical path is illustrated in Figure 16. Forty-eight optical fibers were used to transfer data from the surface control office to the underground shaft station. Then, 24 optical fibers were adopted for underground data transmission. Two intermediate stations, a shaft station and a working section sub-station, were set up, in underground roadway, for data relay. The data transmission distance, from the ground control chamber to the underground monitoring site, was approximately 4100 m. The fiber connections in the optical path require a number of fusions and fiber jumpers, which may lead to a considerable loss of light energy in the optical path. Thus, before the sensors are connected, the optical pass with the smallest loss should be selected experimentally using visible laser radiation and a 10/100 M single-mode fiber transceiver.

### 3.3. Analysis of Monitoring Results

#### 3.3.1. Roof Displacement Monitoring

**Figure 17:**
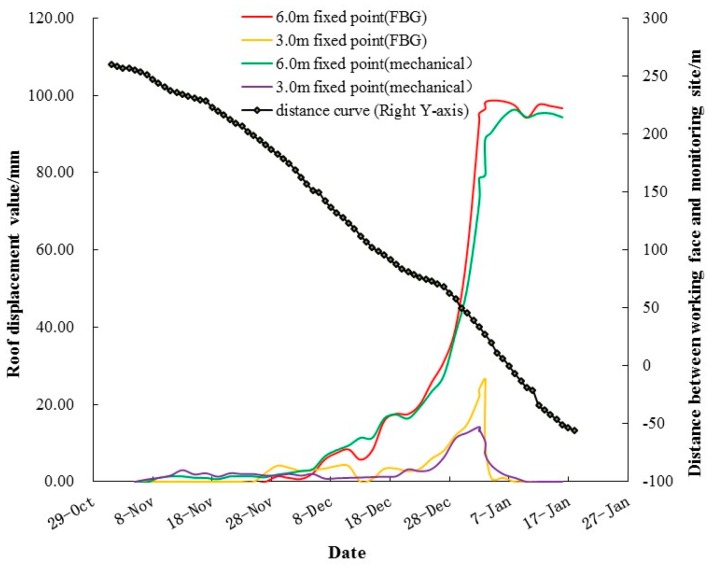
Monitoring results of roof displacement during the approaching of the 1111(1) longwall face.

Figure 17 shows the cross plot of the roof displacement monitoring results and longwall face advances against time (after 80 days of monitoring). Monitoring results from both FBG sensors and mechanical extensometers are shown and the results from these two different monitoring approaches are in good agreement with each other.

The dynamic stress behavior induced by the approach to the longwall face was clearly observed by the FBG sensors. Mining disturbances were first detected by the deep sensors when the face was 150 m away from the monitoring station. This is suggested by the steady deformation of deep roof rock, ranging from 3–6 m. The shallow roof rock (0–3 m) was relatively stable until the face was 85 m away. For both deep and shallow FBG roof separation sensors, most of the roof displacement occurred when the 1111(1) longwall working face advanced from 71 m to –3 m. After the working face passed the monitoring station, the reduction of vertical displacement in the shallow roof rock indicated the re-compaction of rock in this range. In comparison, no further movement of deep roof rock was observed, which indicated an overall spacing between the shallow and deep roof rock of 100 mm. In addition, most of the roof subsidence occurred within 3–6 m of the roadway, which can be a deformation as large as 80 mm. This is probably due to the fact that this range of roof rock is outside the effective radius of the bolting system, which is normally 2.5 m, depending on bolt length. Special treatments, including enhanced cables, long bolts, and grouting targeted at this range of roof rock, are currently being considered in order to reduce the large roof subsidence at the mine.

Although mechanical extensometers and FBG-based roof separation sensors share comparable monitoring results with respect to roof displacement, FBG-based roof separation sensors provide an approach that avoids arduous and frequent underground manual labor. Once installed, FBG sensors can provide automated data collection and transmission. Furthermore, the real-time continuous data sampling performed by FBG sensors grants onsite engineers the ability to assess roof stability and to reduce the risk of roof failure within the shortest time.

#### 3.3.2. Normal Stress Loading on Roof Bolts

**Figure 18:**
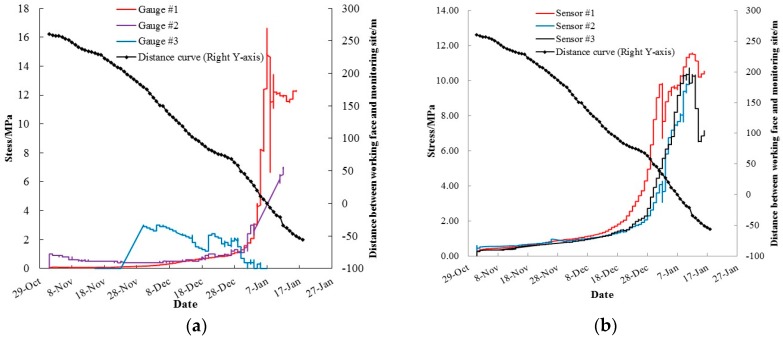
Monitoring results of normal stress loading on roof bolts during the approaching of the 1111(1) longwall face: (**a**) Electric pressure gauges; (**b**) FBG stress sensors.

The observed changes in the normal stress loading of roof bolts, and the distance between the working face and the monitoring station, against time, are shown in Figure 18. Measurement results from traditional electronic gauges are highly divergent, depending on the type of gauge, while a good consistency can be obtained from the results provided by FBG sensors. The high divergence can be attributed to the fact that traditional electronic gauges are sensitive to the harsh underground environment, in which vibration, humidity, or temperature can easily undermine measurement results. In addition, data provided by FBG sensors indicate a much smoother stress increase, induced by the approach of the 1111(1) working face, which is far more reliable, robust, and consistent when compared to electronic gauges. Therefore, only the measurement results from FBG sensors were considered to be reliable for further analyses.

A dramatic increase of mining-induced abutment stress can be observed during the period where the face moved from 72 m to –5 m. A stress increase of nearly 12 MPa was observed over this period, which set the reference stress for bolt design for succeeding longwall panels. It is not surprising this is almost the same period of time as the largest roof displacement. The above-mentioned monitoring results suggest that the mining abutment stress in the mine can reach as far as 150 m ahead of the coal face. Effective control measures to reinforce roadways and reduce roof subsidence should be implemented within 70 m of the face.

## 4. Conclusions

A novel monitoring system for roof safety control in underground coal mines using FBG sensors has been developed. This system can be used to conduct accurate, reliable, and continuous online monitoring of roadway roof activity, with the help of FBG-based roof separation sensors and stress sensors, which facilitates the prevention of catastrophic roof collapse accidents. The proposed system was successfully applied in a deep, hazardous roadway at Zhuji Coal Mine, China. The dynamic impact of the progressive face advance on roof displacement and stress was accurately captured, which provided essential information in order to guide and optimize roadway support design at the mine.

Compared with traditional monitoring instruments, the FBG-based monitoring system has a number of distinct advantages. Although mechanical extensometers and FBG-based roof separation sensors share comparable monitoring results with respect to roof displacement, FBG-based roof separation sensors provide an approach to avoid arduous and frequent underground manual labor. In addition, the continuous and real-time data collection can allow mine operators to remediate potential hazards with the shortest response time. Compared with the electronic gauges in monitoring bolt loading, FBG-based stress sensors are far more reliable and consistent in measuring the stress response during the working face approaching.
